# “The heart in a bag”: The lived experience of patient-caregiver dyads with left ventricular assist device during cardiac rehabilitation

**DOI:** 10.3389/fpsyg.2023.1116739

**Published:** 2023-03-10

**Authors:** Giada Rapelli, Emanuele Maria Giusti, Silvia Donato, Miriam Parise, Ariela Francesca Pagani, Giada Pietrabissa, Anna Bertoni, Gianluca Castelnuovo

**Affiliations:** ^1^Department of Medicine and Surgery, University of Parma, Parma, Italy; ^2^Psychology Research Laboratory, Istituto Auxologico Italiano IRCCS, Milan, Lombardy, Italy; ^3^Department of Psychology, Catholic University of the Sacred Heart, Milan, Italy; ^4^Department of Humanities, Univerisity of Urbino Carlo BO, Urbino, Italy

**Keywords:** left ventricular assist device, heart failure, caregiver, dyad, lived experience

## Abstract

**Objective:**

The Left Ventricular Assist Device (LVAD) has increasingly become a primary therapeutic option for longer-waiting heart transplant lists. Although survival rates are growing, the device requires complex home care. Therefore, the presence of a caregiver trained in the LVAD management is important for the success of the therapy. The LVAD leads both patients and their caregivers to experience new challenges and adapt to new lifestyle changes and limitations – but their subjective beliefs before home management remained little explored.

**Design:**

This study identified, using a phenomenological hermeneutic approach, the main components of the LVAD experience of six patient-caregiver dyads interviewed during cardiac rehabilitation.

**Results:**

We identified 4 master themes: *Being between life and death, Being human with a heart of steel, Sharing is caring (and a burden), and Being small and passive*.

**Conclusion:**

The knowledge from this study can be used as a guide for healthcare providers in counseling LVAD recipients and their caregivers.

## Introduction

In the last decade, the left ventricular assist device (LVAD), a modern electromedical circulatory support used in patients with end-stage heart failure (HF), has increasingly become a primary therapeutic option for longer-waiting heart transplant patients or for those patients who are not eligible for transplantation (Allen et al., 2012).

Although LVAD has been shown to increase survival rates (Smith and Franzwa, 2015), many factors could adversely influence its management, such as device malfunctions, infections, bleeding, and stroke. For LVAD therapy to be successful, guidelines recommend having strong social support and a designated primary caregiver to assist with post-LVAD care (Cook et al., 2017).

Furthermore, LVAD dramatically changes the lives of patients (Modica et al., 2015; Abshire et al., 2016; Bidwell et al., 2017) and their caregivers (Cicolini et al., 2016; Rossi Ferrario and Panzeri, 2020), requiring them to cope and adapt to several challenges related to both the device (e.g., changing power sources or resolving troubleshooting alarms) and daily activities (e.g., bathing, dressing, sleeping, home management, work, and driving).

As a consequence, the psychological distress of patients with LVAD and their caregivers increases. Mainly, symptoms of anxiety, depression, decreased quality of life, and posttraumatic stress disorder (PTSD) are usually experienced from pre-implantation to 3–6 months post-implantation in both patients and their caregivers (Bunzel et al., 2008; Brouwers et al., 2015). Although the management of LVAD is more complex, the psychological adjustment of patients and informal caregivers could be comparable to that experienced with the Implantable Cardioverter Defibrillator (ICD; see for a comparison Saunders, 2017), in fact, both LVAD and ICD are life-saving cardiac devices and in both cases patient-caregiver dyad has to cope with new challenges in the course of physical healing, including knowledge of a device, the stress related to fear of reduced self-sufficiency and to feel a burden to their families (Saunders, 2017). Importantly, patient outcomes and those of their caregivers are typically interdependent, as characteristic of dyadic processes in intimate relationships. This means that the outcomes of one member of the dyad influence the outcomes of the other member and vice versa, as shown in studies involving the non-clinical population (e.g., Donato et al., 2015), patients with cardiovascular problems (e.g., George-Levi et al., 2016; Rapelli et al., 2021, 2022a), and with LVAD specifically (Bidwell et al., 2018; Rossi Ferrario and Panzeri, 2020).

Specifically, research that examines the outcomes of both patients and their caregivers could better estimate how they experience and manage the illness together (e.g., Bertoni et al., 2015).

To summarize the extant literature on patients and caregivers with LVAD, two main themes were identified:

- How did patients and caregivers make decisions (together or not) throughout the course of the illness? (e.g., McIlvennan et al., 2018; Birriel et al., 2019);
- How was the psychological well-being of patients and caregivers before and after LVAD implantation? (e.g., Brouwers et al., 2015; Bidwell et al., 2017).

Despite extensive research on these issues, there is a gap in the literature regarding the thoughts, fears, expectations, and hopes of patients and caregivers during cardiac rehabilitation (CR), before discharge and the return home. In fact, in this period, the worries related to the survival of the invasive intervention are fading, and people start to have different concerns, especially those related to the home management of the device. At the same time, the success of the intervention could generate feelings of hope for the future and for the expectation of the new heart. In fact, as has already been demonstrated in cardiac patients, the CR could serve as a vehicle for motivation and persuasion as well as a critical point for tracking the cardiac patients’ quality of life, in both the presence and absence of significant psychological distress (Pietrabissa et al., 2017; Spatola et al., 2018, 2021).

Given these gaps in literature, the present study was aimed to explore the lived experience of patients with LVAD and their caregivers before discharge from the hospital, using a dyadic phenomenological hermeneutic research design, in order to identify their needs and concerns related to this important and delicate life transition and the preparation of home care tasks. This dyadic approach allowed us to identify the impact of the illness on both members of the dyad exploring their reciprocal interdependence and giving voice to both the recipients of the cure and their caregivers, describing their personal experiences and analyzing similarities or discrepancies in the narratives.

## Materials and methods

### Study participants

A purposive sample of 6 patients with LVAD and their caregivers (for a total of 12 individual interviews) were enrolled from Istituto Auxologico Italiano, Ospedale San Luca, Milano, an inpatient cardiovascular clinic in northern Italy.

Patients were selected for inclusion into the study at admission to the program according to the following inclusion criteria: (1) being implanted with the HeartMate III LVAD as a bridge to transplant (BTT), (2) being in a CR program on average 1 week after LVAD positioning, (3) aged 18–65 years, and (4) being Italian speaking. Selected inclusion criteria for caregivers were, instead, being LVAD close relative that lives with the patient and speaking Italian. They were typically the closest related family members.

All patients and caregivers approached for the study agreed to participate.

The patients were all male with a mean age of 48, 79 (SD = 8.55) and an age range of 39–64 years. Their level of education is moderate to high. Five out of 6 patients had a cardiological history between 7 and 12 years with a diagnosis of post-infarct dilated heart disease and had been on the transplant list for an average of 4 years. Only one patient was a candidate for LVAD following an infarction with late hospitalization. Regarding the proximate pathological history, all patients were admitted for advanced heart failure often with renal and hepatic damages. Given their age and the difficulty of receiving a transplant, an LVAD was implanted as a bridge to candidacy following collegial discussion. Two of them were single, 1 was divorced, and 3 patients were married. Caregivers in the sample were 3 wives, 1 mother, 1 brother, and 1 cousin. The mean age of the caregivers was 51.11 (SD = 8.77) and their ages ranged from 49 to 71 years (Table 1). The caregivers all have a high school education.

**Table 1:** Demographic characteristics of participants in the study.

Participant	Patient age (year)	Patient sex	Educational level	Work occupation	Marital status	Designated caregiver	Caregiver age (year)	Children
#1	64	Male	Degree	Retired	Single	Cousin	56	–
#2	39	Male	High school	Employed	Single	Mother	67	–
#3	56	Male	High school	Employed	Divorced	Brother	53	–
#4	57	Male	High school	Employed	Married	Wife	49	1
#5	59	Male	High school	Occupied	Married	Wife	71	–
#6	58	Male	Junior high school	Unemployed	Married	Wife	53	1

### Procedure

Data were collected from June 2018 to June 2019, until data saturation was achieved. In general, the dyads brought similar themes, and continuing with the interviews, the latest dyads did not bring any new themes to those that had already emerged and so data saturation was reached (Morse et al., 2002; Silverman, 2010).

Using a phenomenological hermeneutic research design (Lindseth and Norberg, 2004), a trained psychologist (first author^1^) conducted a face-to-face individual semistructured interview with patients with LVAD and their caregivers during the CR program.^2^ Each interview was conducted separately so that patients and caregivers could provide their own points of view without being influenced by the other (Ummel and Achille, 2016).

Before the interview began, the interviewer informed the participants about the aim and procedure of the study and obtained their signed informed consent. With informed consent, patients and their caregivers also gave professional permission to audio record the interview.

Individuals were made clear that their participation was voluntary, and that they could quit the interview at any time. Participants did not receive any type of reward for study participation.

None of them decided to leave the study during the interview.

The interview occurred in a dedicated room of the hospital and lasted from 30 min to 1 h. No one was present in the room beside the participant and the psychologist.

The semi-structured interview focused on the experience of an implantable device, feelings, and worries about LVAD management, feelings about heart transplants, and heart donation.

A storytelling approach with probing questions (such as *“tell me more about that experience”* and *“how did that make you feel?”*) was further used during the interview to clarify or expand meanings presented by the participants – thus facilitating a dialogic interaction process. This is fundamental while conducting a phenomenological study, to help participants to express their deepest thoughts and feelings as freely as possible (Eatough and Smith, 2008).

The interviews ended when participants had nothing more to add. The interviewer did not field notes during or after the interview.

A demographic profile (age and level of education) of the interviewed was obtained at the end of each interview (for details see Table 1). After interviewing six dyads (six patients and six caregivers), data saturation was achieved. In line with the literature, phenomenological studies typically achieve data saturation with 10 or fewer participants (Dukes, 1984). Moreover, the caregivers were 3 partners and 3 other family members (mother, brother, and cousin). This triangulation of data, with comparisons of different provider profiles, allowed us to capture the essence of the LVAD experience.

The study was approved by the Ethics Committee of the [*removed for review process*]. All procedures were run in accordance with the 1964 Helsinki Declaration and its later amendments.

In this study the consolidated criteria for reporting qualitative research (COREQ; Tong et al., 2007) was followed (Supplementary File 1).

### Data analysis and rigor

The interview transcription data were analyzed qualitatively using a phenomenological hermeneutic research design (Lindseth and Norberg, 2004). The transcriptions were not returned to participants for their feedback. A data-driven approach (versus theory-driven) was used for text analysis, and given the analytic approach was inductive, the comparison with the research literature was conducted only at the end of the whole process as a sort of “return to the theory” (Lindseth and Norberg, 2004). All emerging themes were derived from the words of our participants, rather than from preconceived theoretical concepts and published research evidence; furthermore, the data-driven approach has been considered the most effective way to investigate subjective experiences (Davidson et al., 2008). Two authors independently performed data analysis without using any software. Disagreements between coders were reconciled through extensive in-person discussion. The findings were constantly shared and discussed with the whole multidisciplinary team, which enhanced the researchers’ reflexivity and thus reduced the influence of their preconceptions and biases on the analytic process, with increased rigor. The analytic process initially entailed line-by-line reading of each interview to provide a preliminary description of relevant topics, with notes recorded directly in the text. The interview, in a primary recursive phase, has been read and re-read, primarily for each participant, and then across participants with constant attention to the connection between each interview and the whole corpus of interviews. In fact, with each reading, new insights and conceptual aspects dense with meaning emerged. This circular process led to the identification of a set of superordinate themes and subthemes when similarity/discrepancy emerged. During this process, some themes were dropped, for example those that did not fit well with the emerging framework or those that were less well represented and had less evidence in participants. The goal was to make sure that the analysis matched the participants’ accounts and that each account presented was justified by the data.

To establish the trustworthiness of our findings, we triangulated the patient and caregiver data to explore the lived experience of LVAD from multiple perspectives (Bekhet and Zauszniewski, 2012).

In the absence of specific guidance on how to analyze qualitative data dyadically (Ummel and Achille, 2016), analyzes were performed following the three recursive methodological steps described by Lindseth and Norberg (2004), beginning with the individual analysis of the data provided by the member of the dyad who had been interviewed first, followed by the analysis of the data provided by the other. Since the aim of the study was to explore the dyadic experience of living with LVAD for patients and their caregivers, this dyadic approach provides evidence of the experiences of both members of the dyad, the differences between each participant’s experiences, and their points of divergence and convergence. Shared themes, connections, and discrepancies between patients and caregivers were identified. Dyadic congruence was defined as a shared understanding or aligned perspectives regarding a particular topic between the patient and the caregiver. First, we compared the patient group with the caregiver group to see whether the two shared a perspective overall. Then, each dyad was examined to explore within-dyad congruence regarding a topic. The congruence for each major theme was examined with independent evaluations by each coder followed by discussion and consensus during team meetings.

## Results

The text analyzes identified four dominant themes that characterized the lived experience of patients and their caregivers with LVAD: (1) Being between life and death, (2) Being human with a heart of steel, (3) Sharing is caring (and a burden), and (4) Being small and passive. Each theme involved a set of subthemes, which were considered as further specifications of the meaning conveyed by the dominant themes and were obtained by identifying similarities and differences between interviews within themes. Dominant themes and subthemes are reported in Table 2. The frequency of themes derived from the analysis is illustrated in Table 3.

**Table 2:** Dominant themes, subthemes, and explanation.

Dominant themes	Subthemes	Explanation
1. *Being between life and death* Patients and caregivers are on a fence. They live ambivalent experiences of life, acceptance, hope and death, pity, pessimism. These experiences are prior to the implantation of the LVAD, but also subsequent.	*Being on a roller-coaster*	A life of ups and downs, from hardships, repeated hospitalizations, renunciations and sacrifices, but also moments of hope and optimism. These experiences are reported both before the LVAD implantation when the patient and the caregiver are engaged in the management of the symptoms of HF or even after the implantation in those dyads who still perceive moments of fatigue and sudden changes.
	*Being between denial and acceptance*	Accept the LVAD with resignation, without alternative, because the alternative is to die. On the opposite side of the binomial we find instead rejection of the device, contempt, openly hostile behavior that is recognized verbally, but sometimes even unconsciously, for example by strangling the LVAD.
	*Living the post-LVAD like a bridge to heart transplant or an ordeal*	The moment after the implantation of the LVAD that patients are living in the hospital and that they are facing with respect to home management can be perceived by the patient and the caregiver as a bridge to the real goal, which is transplantation or, on the contrary, as a tiring and distressing moment full of changes and challenges, even uncertain ones.
2. *Being human with a heart of steel* This issue raises the question of the heart not only as an organ, but also as a seat of emotion and soul. Patients and caregivers talk about the fears of managing a device and all the complications that come with mismanaging it. Having an artificial heart also reframes one’s definition of being human	*Being afraid of the unknown*	Patients and caregivers show fears and uncertainties about the future and management of LVAD. They have concerns about the home management of the machine and its care (alarm management, maintenance, wound and driveline care and medication), but also fears about the uncertainty that such a change in life can create, for example in family and social relationships.
	*Becoming part of the machine: The embodying process*	Patients and caregivers come to consider the machine as embodied, as part of the body or as an extension of it. This, on the one hand, suggest a process of acceptance, on the other hand, could represent a risk of excessive dehumanization, in which patient perceives himself (or is perceived) as a cog-like machine, emphasizing a part-whole problem.
	*Having a heart, not only a pump!*	Having a machine instead of a heart awakens in patients and caregivers the symbolic-emotional aspect of the heart. Participants also problematize the question of treating the person not just the body, as in an integrated mind–body approach.
3. *Sharing is caring (and a burden)* In this section there is the theme of care. The presence and help of a caregiver is perceived as indispensable, not without consequences in the patient who feel himself/herself like a burden and in the caregiver who could experience burnout syndrome due to the burden of care and lack of adequate psychological support.	*Being two is better*	Patients recognize the importance of dealing with the LVAD implantation and its management with someone who can help them manage not only the device, but also the emotional and psychological aspects that follow. Caregivers also recognize themselves as central figures for handling the situation from a practical and supportive point of view.
	*Feeling like a ball and chain*	Patients often feel like a drag on their loved ones because of the burden of care required by the caregiver. Caregivers themselves recognize that patients often have this sense of dependence on them that makes them feel less autonomous
	*Feeling like a drained battery*	Caregivers perceive a burden of care that sometimes follows caregiving for years of chronic heart failure. Caregivers are concerned that they will not be able to cope psychologically with the situation they are facing. This burden and the ensuing worry is also a concern of the patients. Caregivers feel fatigued by the burdens of care, as if they were without batteries.
4. *Being small and passive* In this last section we deal with ethical and existential issues. A general sense of smallness and passivity emerges. The situation is in fact recognized as the will of God or a scientific power over which man has little autonomy and sense of will, but only passively receives the machine, waiting (even more passively) for the arrival of a suitable heart.	*Being grateful to God*	Although the situation is delicate, those who have faith, but also those who have never had it, feel grateful to God for the opportunity, for a second life.
	*Being against nature*	The presence of a mechanical part brings out in the participants (both patients and caregivers) a feeling of being counter-nature, a perception of non-human immortality that creates anguish and questions about the meaning of life and its finiteness.
	*Waiting for the* *heart donation: When is the gift given?*	Waiting for a heart with long waiting lists is not easy for patients and their caregivers, and they wonder if this “gift” will come sooner or later. The situation of organ donation in Italy is also problematic.

The original language of the interviews was Italian. English translation of quotations was performed by the first author and checked for accuracy by all authors. Subsequently, the English translation has been confirmed by an external English reviewer. In order to increase rigor, all the meaning of thematic categories is discussed and confirmed by all authors.

**Table 3:** Frequency of subthemes identified with interviews.

	Dyad 1		Dyad 2		Dyad 3		Dyad 4		Dyad 5		Dyad 6	
	Pt	Crg	Pt	Crg	Pt	Crg	Pt	Crg	Pt	Crg	Pt	Crg
Being on a roller-coaster	✓	✓				✓	✓	✓		✓		✓
Being between denial and acceptance	✓	✓	✓	✓	✓	✓	✓	✓			✓	✓
Living the post-LVAD like a bridge to heart transplant or an ordeal	✓		✓	✓	✓	✓	✓	✓		✓	✓	✓
Being afraid of the unknown	✓	✓		✓		✓	✓	✓	✓		✓	✓
Becoming part of the machine: The embodying process		✓	✓		✓	✓	✓		✓	✓	✓	✓
Having an heart, not only a pump!	✓		✓			✓					✓	✓
Being two is better		✓	✓		✓	✓		✓		✓	✓	✓
Feeling like a ball and chain	✓			✓	✓							✓
Feeling like a drained battery	✓	✓		✓		✓	✓	✓			✓	
Being grateful to God		✓		✓	✓				✓	✓	✓	✓
Being against nature	✓				✓			✓	✓		✓	
Being waiting for the heart donation: When is the gift given?	✓					✓	✓		✓	✓	✓	✓

Pt, Patient; Crg, Caregiver.

### Being between life and death

Contrasting feelings characterized both patients’ and caregivers’ lives in pre-and post-LVAD implants. In fact, their lives before LVAD were precarious due to symptoms of HF and the only solution was the LVAD implantation; but this choice was made with ambivalence. Patients and caregivers reported that this initial phase of adjustment to LVAD was like being on a rollercoaster, wavering between denial and acceptance and seeing the LVAD as a solution but also as an ordeal. These themes highlighted the fine line between life and death they experienced following LVAD implantation.

#### Being on a roller coaster

Life before LVAD was characterized as having sudden hospitalizations for HF with debilitating symptoms such as shortness of breath, fatigue, and pain. This period of precarious health was variable and could last for years, with the unpredictable ups and downs having a substantial impact on psychological well-being.

*“Climbing stairs involved heart fatigue, tying shoes… Trivial gestures in everyday life are not normal in heart failure, brushing your teeth causes heart fatigue.” (patient #1)*

*“It’s been 10 years from hell. We did not sleep at night. There were very hard moments. He was constantly hospitalized for heart failure. He was having difficulty breathing, he weighed 130 kg. His heart worked 20%.” (caregiver #6)*

The time before the implantation of LVAD was reported by patients and caregivers as very difficult and characterized by multiple daily-life challenges. The demands associated with living with an HF imposed physical and emotional demands on both patient and caregiver. Caregivers also reported that this period had a significant impact on their quality of life.

Likewise, the perception of life after the LVAD implantation was similar to that experienced before implantation; In fact, the post-LVAD phase was characterized by frequent abrupt and unpredictable changes, like being on a rollercoaster. Participants described stressful and tiring periods followed by more peaceful and adjusted moments.

*“It’s still a machine, it can go right or wrong.” (patient #1)*

Although feelings of fear and concern about what could happen emerged, a sense of trust, hope, and optimism for the future and for a cure also was observed.

*“I look at who is worse, if they can do it, we can do it very well too.” (patient # 1)*

*“This is the solution. You have to take the pros and cons. But at least there is something!!! We live in a world where disease is around us and, in some cases, there is no cure. Here there is a great disease, but there is also a great hope of cure and recovery.” (caregiver #3)*

After a period of uncertainty and vulnerability, despite the efforts and difficulties in managing firstly the HF and suddenly the LVAD – also because the LVAD option was proposed when medications typically could no longer compensate for the decline of heart function and patients had only this therapeutic option – feelings of hope and trust allowed the patient to accept the device and the caregivers to move forward in their role. These mixed feelings underlined the ambivalence that patients and caregivers had in the initial phase of LVAD. The perception of being on a rollercoaster was reported mostly by caregivers (3 out of 6), but 2 dyads shared the same opinion on this initial phase.

#### Being between denial and acceptance

The phase of pre-operative evaluation of patients as candidates for LVAD implantation flooded patients and caregivers with feelings of uncertainty, vulnerability, and loss of individual psychological integrity. In fact, at this stage, patients were informed about the benefits and risks of the device, and the clinical reasons for choosing the LVAD were discussed. This waiting period was, therefore, full of anxiety, fears, and expectations. Still, in some circumstances, due to the worsening of clinical conditions, the intervention was carried out on an emergency basis as a forced choice, times were very tight and this could compromise the patient’s psychological state with symptoms of anxiety, confusion, and fear.

*“I have a relationship of love and hate with this device. I know, it saved my life and allowed me to do the stairs at home. However, it is a technology that is a bit obsolete, a bit intrusive.” (patient #1)*

*“We did not believe that it would come to this point, because he still does not accept it. I have been taking sedatives every day for ten years, and even more after implantation. I probably do not accept it any more than he does.” (caregiver #4)*

Patients and caregivers said during the interviews that the fear often was so great that it was accompanied by a feeling of denial. At the same time, having no other options, patients and caregivers were compelled to accept change and the device, because the other option was to die. A feeling of ambivalence emerged: participants reported that they had to get used to the device and had to accept it. All participants, and 5 dyads out of 6, shared the same perception of LVAD as a valid option, but one that was difficult to manage and accept. The caregivers also showed mixed feelings about the LVAD option. Still, a general acceptance of the device and gratitude for it was present because it allowed loved ones to survive.

*“I am sorry to see him like that attached to a device, but at least he is here!” (caregiver #2)*

Specifically, it seemed that feelings of withdrawal gave way to feelings of acceptance.

#### Living post-LVAD like a bridge to a heart transplant or an ordeal

The feeling of ambivalence returned in both patients and caregivers when thinking of the management of LVAD. In fact, LVAD was appreciated for the advantages it conferred, such as decreased HF symptomatology and increased life expectancy, but at the same time, it required a period of adjustment as new roles, and relationship changes had to be negotiated within the high-stakes context of LVAD care. For some participants, the LVAD could be a bridge to heart transplant for some others the adjustment was considered stressful and as an ordeal.

*“Now I see the finish line there, it is near.” (patient #2)*

*“An ordeal awaits us. One day he will be well, one day he will be bad. The life that awaits him will be like this, hospitalization, infections. For the next 6 months or 7, he will not even be on the list for a transplant anymore. It will be an ordeal; it will be a thing to be experienced step by step. We must live in the present.” (caregiver #3)*

Patients and their caregivers had diverse visions for their journey to the LVAD: Some participants, both patients, and caregivers, considered the LVAD a first step to the heart transplant, triggering the idea of a long journey aimed at obtaining a new heart; others viewed the LVAD period as an ordeal with many threats to overcome. This feeling concerned the whole family. These themes emerged in 4 dyads and 1 other caregiver who shared the same perception about LVAD management.

### Being human with a heart of steel

The presence of an artificial heart raised fears, some typically human such as fear of the unknown as well as some not typically human such as fear of power cuts that would cause problems with the LVAD. Likewise, patients and caregivers wanted to point out that despite the presence of an artificial heart, you remain human, with feelings and emotions, alluding to the heart as a symbolic seat of emotions.

#### Being afraid of the unknown

When a patient with LVAD comes back home, changes in lifestyle, home environment, habits, and even roles are needed. The management of the LVAD requires constant assistance and knowledge of all guidelines for device malfunctions or procedures to undertake if the patient feels sick.

*“I am afraid to go among people, to be jerked and the driveline will come off and my heart will stop. I am afraid that my briefcase will be stolen, maybe someone will think it’s something of value.” (patient #6)*

*“I am afraid there is no electricity, it is a constant worry.” (patient #1)*

Patients were worried about home management of the LVAD and possible complications like infection or stroke. Fears also emerged concerning their social life. In fact, patients were worried that the LVAD might suffer damage caused by involuntary bumps. The patient also noted a constant fear of power cuts, since the LVAD requires its batteries to be recharged during the night.

The caregivers also showed the same concerns as patients with anxiety, because both patients and caregivers had to adjust to a new set of life circumstances and task demands related to managing LVAD-related care (e.g., driveline dressing changes, maintaining a power supply, and reporting device-related complications).

*“We have to learn how to manage everything, the batteries, the alarms, then he will need 24-hour assistance because if he feels bad and the battery stops, I will have to call for help.” (caregiver #2)*

Caregivers felt overwhelmed by the huge responsibility and did not yet feel fully prepared to manage it. In addition, caregivers were worried about performing a sterile dressing; in fact, the risk of complications due to catheter-related infections was usually high among patients with LVAD.

The changes required by the LVAD involved the whole family. These themes related to LVAD management emerged in 4 patients and 5 caregivers, and 3 dyads shared the same perceptions.

#### Becoming part of the machine: the embodying process

In many cases, patients and caregivers began to consider the device and the person as one, in a sort of embodying process. This aspect requires specific attention since not recognizing the presence of the device, for example, tugging it or trivializing its management, could lead to serious consequences.

*“He has already told me that if he is comfortable with the LVAD, he does not want to have the transplant, so in the end he has nothing, he is fine before he was attached to the machines, but now he is walking.” (caregiver #6)*

*“He is like a bionic man.” (caregiver #3)*

As a negative consequence of this process, the patient could fantasize that he could do everything with LVAD, forgetting he had it and engaging in risky behaviors. For example, the patient could refuse a transplant due to the “magic” and positive outcomes of the device, as one caregiver reported above. Likewise, the caregiver could perceive the patient as healthy because the LVAD is hidden under the skin and is not visible to others. This could be considered either a process of full acceptance of the device or a process of trivialization or denial and dehumanization performed not only by caregivers but also by patients themselves. Furthermore, it could influence the definition of self and his/her own identity; in fact, the LVAD contributes to making the person seem less human. These themes emerged for 5 patients and 4 caregivers, and 3 dyads shared the same perception.

#### Having a heart, not only a pump!

Patients and caregivers reported that such an intervention involving a major life change required addressing its related psychological and emotional aspects. Often participants did not think of the heart as a mere organ, but had a symbolic vision of it-as the deepest part of themselves and the seat of their emotions.

*“To the doctor who told me that I had an enlarged heart due to heart failure and I had to put in the LVAD, I replied: I do not have an enlarged heart, doctor, I have a big heart.” (patient #6)*

*“He needs support, someone to talk to because it’s not that the operation went well then everything is ok, now we need to reassure him.” (caregiver #6)*

In the early stage of LVAD, patients and caregivers considered the importance of being supported psychologically. They had the idea that the intervention involved not only the body, but also the mind, their emotions. These themes emerged in 3 patients and 2 caregivers, and 1 dyad shared the same perception.

### Sharing is caring (and a burden)

With respect to the care relationship that patients inevitably had with their caregivers, which would sometimes turn into a relationship of dependency due to the need for constant and prompt care, both the patients and partners recognized the indispensable presence of the caregiver, but in the same way, they recognized aspects of burden: patients feared to be a burden for their loved ones and caregivers feared stress from the care load.

#### Being two is better

The caregiver role was perceived as important for patients at the early stage of the treatment. They recognized that being two in making decisions and managing the device, as well as having the opportunity to share thoughts and fears, could make a difference in living with LVAD.

*“Even the doctors told me that being two is better” (referring to the wife). (patient #6)*

*“Four eyes are better than two for the complexity of the LVAD management, even now I tell him: ‘Check the batteries, check the driveline…’”. (caregiver #4)*

The LVAD management was challenging and the caregiver needed to maintain a high degree of vigilance to assure patient safety, survival, and the ability to remain out of the hospital. Patients and caregivers perceived that the patient could not cope alone with this device, but this theme emerged mostly in caregiver participants: 5 of 6 caregivers and 3 patients reported the importance of caregiver role; in addition, 2 dyads shared the same perception. Furthermore, participants reported that caregivers provided not only practical but also emotional support; during the critical moments when the patient felt at the mercy of emotions and uncertainty, the presence of the caregiver was considered essential.

*“He did not want to do this intervention, because he was terrified, he told me he preferred to die. I took matters into my own hands and convinced him.” (caregiver #4)*

Some caregivers felt they had a key role in assisting their loved one in the complex management of the LVAD, thus recognizing the importance of following the LVAD’s guidelines, which recommend the constant presence of a trained caregiver.

#### Feeling like a ball and chain

Patients recognized the delicate role played by caregivers and were afraid of being a burden to them to such an extent that they would feel like a ball and chain. In this regard, some patients called themselves an “imposition” and were afraid of having to depend entirely on the caregiver.

*“It weighs me to be dependent on others.” (patient #3)*

*“He feels a burden, but it is 7 years that is like this… I do not know what to say to convince him that he is not a weight.” (caregiver #2)*

Patients felt dependent on caregivers and this perception was also confirmed by caregivers themselves. On the one hand, patients reported negative aspects of caregiving due to lack of mutuality, change in the relationships, burden for caregivers and lack of autonomy for the patients. Caregivers feared hurting the recipients or due to their lack of preparedness, denied the burden of care. This theme emerged in 2 patients and 2 caregivers; no dyad shared the same perception.

#### Feeling like a drained battery

The caregiver role was experienced as a burden by some patients and some close relatives and, in some cases, the caregiver burden was associated with signs of psychological distress. The greatest stressful situations occurred when the caregivers perceived their role as tiring and poorly supported during the years preceding the LVAD implantation. Caregivers were also concerned about having to provide emotional support to their loved ones, an additional task they did not feel totally prepared for, especially because they themselves were distressed to such an extent as to feel like a drained battery.

*“I am tired, I am depressed, I cannot take it anymore, and with this LVAD it will be worse and worse … If he is in high spirits, he is serene, that’s fine, otherwise I can’t do it.” (caregiver #4)*

*“You can't always be thinking about whether the LVAD battery is charged or discharged, otherwise then I'll discharge myself, and it's over!” (caregiver #3)*

Patients and caregivers reported that the caregiver’s life was characterized by perceived stress, depression, role strain and burden. This situation was caused by the intensity of care before LVAD as well as the management of the device at home. The perceived strain was also a consequence of the loss of patient autonomy. Furthermore, the caregivers’ psychological distress could be due to the excessive apprehension and solicitous care that is required, also called overprotection. In addition, one caregiver metaphorically used the image of CPAP batteries to refer to the caregiver burden as a person with dead batteries who has been exhausted by the illness itself. This theme emerged in 2 dyads and 2 other caregivers perceived themselves like a drained battery.

### Being small and passive

A feeling of fragility and human smallness emerged in both patients and their caregivers. The implant was represented as a rebirth attributed to either God or science, for which patients and caregivers felt grateful, but at the same time, they had ambivalent feelings to the point of feeling “against nature.” Even the wait for a transplant was lived with passivity, a weary wait during which patients and caregivers have expectations, but also an absence of mastery and control of the situation that makes them feel helpless about the future.

#### Being grateful to god

The LVAD is a significant life change for those who receive it. It provides a better quality of life after the suffering and pain experienced with HF symptoms. Some patients and their caregivers attached spiritual significance to this medical option. The psychological quality of life along with the spiritual quality of life of the patient and his or her family were important targets to be assessed in the first phase of adaptation to the device. However, patients who attribute a spiritual meaning to the implantation of the LVAD experience a sense of passivity, as if this choice was not theirs to make, but was chosen from above, by someone greater than themselves.

*“I thank God that this device is there, otherwise I would die. It is a burden, a sacrifice, but thank goodness it is there.” (patient #5)*

*“The faith in God is all you have left, when your life is hanging by a thread.” (caregiver #5)*

Patients and their caregivers unanimously described the experience of the LVAD as an opportunity for their spiritual life. They attributed their living and the presence of the device in their life as the will of God. Spirituality was also viewed as a coping strategy when life was hanging by a thread (symbolically the power line of the LVAD) as mentioned by a caregiver. Those who had this vision were also more positive about the future with LVAD and its management. This theme emerged in 3 patients and 4 caregivers; 2 dyads shared the same perception.

#### Being against nature

Often, as emerged from the previous themes, the LVAD was a forced medical option decided by others (either physicians or caregivers) *in extremis*, and this could influence how patients or their caregivers accepted the device and gave it a positive meaning with a consequent better psychological adaptation and quality of life. Sometimes, instead, those who received the LVAD showed resistance and reported that this choice wanted by others conflicted with their personal values, so much so that they felt they were no longer human because they were attached to a machine that was keeping them alive.

*“It makes me feel strange to think that if it had been for God I would have died, science in some ways defies life.” (patient #1)*

*“He said to me: ‘When I die, I die, I do not feel like doing this thing against nature”. (caregiver #4)*

For some patients and caregivers, the LVAD implantation was not in line with their personal values. In fact, some participants perceived the device as a constraint preventing them from living, almost as an “aggressive medical treatment” that prevents nature from taking its course. This theme also prompted reflection on how patients and caregivers define life and quality of life and the importance of informing recipients of what it means to live with LVAD, and whether this choice takes into account their preferences regarding end of life, definitions of quality of life, and meaning in life while managing the uncertainty of living with LVAD. This theme emerged in 4 patients and 1 caregiver; no dyad shared the same perception.

#### Waiting for the heart donation: when is the gift given?

The LVAD as a bridge to transplantation also prompted reflection on the topic of donation. Patients and caregivers reported uncertainty and fear about this last step, recognizing that the implantation of the LVAD was only the beginning of a long wait and that this wait can sometimes be in vain because the issues for waiting lists for a new heart are sometimes endless. Patients and caregivers could wait for a heart that will never arrive.

*“The culture of donation is important, but not because it is a noble gesture, but because it is a civil gesture.” (patient #1)*

*“If the heart arrives, “if” must be underlined. Because the heart may not match. It is not known if it arrives or does not arrive, and if it arrives and is fine or arrives and is not compatible. There is always a big question mark. If I could give my heart to him, I would do it, but then I die. If it was a kidney, I had already given it to him. It will be tough.” (caregiver #3).*

Given the long waiting list for heart transplantation, patients considered this a very distant goal. They also recognized that heart donors are lacking in Italy-therefore they live through this waiting period with ambivalent feelings of hope, uncertainty, and fear of not being able to obtain a compatible heart. These feelings were also shared by caregivers, who were supposed to manage the “undetermined” waiting period and the psychological tension that accompanies it all on their own.

A sense of helplessness and lack of control also emerged in the caregivers. This theme emerged in 4 patients and 3 caregivers; 2 dyads shared the same perception.

## Discussion

This phenomenological hermeneutic study provided an understanding of the initial process of adjustment to LVAD in both patients and their caregivers.

By interviewing the patient-caregiver dyad before discharge from CR, several topics concerning LVAD-related lifestyle changes, worries about the LVAD management, caregiver role, and the associated bioethical aspects emerged.

Across the various themes that emerged, patients and caregivers showed conflicting feelings about LVAD implantation and its management and also about transplantation. This suggests that in this phase, immediately after LVAD implantation, patients and caregivers are experiencing inner conflicts of ambivalence with the device and what awaits them. In fact, according to the literature (Rossi Ferrario and Panzeri, 2020; Rossi Ferrario et al., 2022) the implantation represents an opportunity for patient’s life in serious danger, but also a threatening phase requiring to assume stronger personal and familiar responsibilities in order to manage the device and the several related lifestyle changes. The implantation of LVAD interrupts the life narrative of these patients and their caregivers. After a past in which they were completely dependent from hospitals, doctors, and medications, they are now required to actively engage in the management of the disease and to integrate into their everyday life this new medical procedure. Moreover, the LVAD likely changes patients and caregivers perception of the future characterized by waiting for the transplant.

Both the patients and their caregivers reported contrasting feelings that characterize both lives before implantation with symptoms of HF and life after LVAD. In fact, life before implantation was characterized by conflicting feelings because of the physical and psychological impairments due to end-stage HF that forced them to a life of sacrifice for the sudden hospitalization, but also trust in treatment. The complex and unpredictable nature of the disease was characterized by a decline in the functional status of the persons, with increasing severity and frequency of symptoms over time punctuated by periods of unpredictable exacerbation from which patients might or might not recover. Moreover, in the literature it is demonstrated that patients with HF reported significant low mood, emotional strain of feelings of loss, role limitations, and feeling as a burden for their significant ones (Brouwers et al., 2015), concerns also reported by patients with ICD (Kajanová et al., 2017). Due to high caregiving demands, relatives reported emotional distress, restricted social life, and sleep disturbances caused by patients’ sleep apnea, and their use of diuretics (Molloy et al., 2005). This period was also characterized by feelings of hope for the confidence in the therapies and waiting for a new heart.

Life after the LVAD was itself characterized by ambivalent emotions for both patients and caregivers, in fact they presented feelings of now accepting and now rejecting the device. They recognized that LVAD is the unique solution to the heart problem, but they were aware of the limitations and complications with moments of downtime and difficulty. This sense of ambivalence regarding the LVAD as a forced choice between life and death is also confirmed in the literature (Magasi et al., 2019), but this study extends our understanding of the emotional storm that patients and caregivers have especially in the initial phase of LVAD. This underlines the need to welcome, listen to, and manage emotional adjustment of both patients and caregivers, shifting from a “cure” to a “care” approach (Donato and Bertoni, 2018). In fact, the adaptation to the device was not immediate, and its management resulted somehow difficult and all participants reported that adjustment took time.

The ambivalence was also reported by both patients and caregivers regarding the conflicting feelings in front of an artificial heart, a theme never investigated in literature to our knowledge. They presented typical human fears associated with the afraid of the unknown, for example in LVAD homecare. As reported by both patients’ and caregivers’ interviews, LVAD required changes in daily routine, roles and home space. Associated with this theme, various worries about the risk of infection at home emerged (e.g., the caregivers were concerned of having to perform the dressing). Moreover, the patients reported fears of being with others and of social life, because of the special attention required by a carrier of LVAD. Some of these worries were investigated and confirmed in other qualitative studies (Casida et al., 2011; Marcuccilli et al., 2014). Receiving LVAD while waiting for heart transplant was perceived by participants either as a first step of a long journey with positive and trusting feelings, or a period of physical and emotional instability also due to many re-hospitalizations. This reaction with mixed feelings of gratitude, re-evaluation of life, and negative feelings related to restriction of physical activity was previously reported in the literature on patients with ICD (Thylén, 2017) and their caregivers (Saunders, 2017), a comparable experience to the challenges which patient and caregivers are called to face. Moreover, the adjustment to a new lifestyle and LVAD home care, as shown in other studies (Casida et al., 2011; Magasi et al., 2019), represented highly stressful processes for both patients and caregivers. In fact, patients tend to score lower on physical health status as compared to their caregivers, but not always on mental health status (Casida, 2005). Furthermore, patients and caregivers scored lower on mental health compared to the general population (Brouwers et al., 2015). In addition to the psychological distress in patients and caregivers, another psychological aspect that emerged from the interviews was the embodying process, detected both in the patients toward themselves and in the caregivers. From a psychological point of view, this relevant theme, never addressed in the existing literature to our knowledge, requires specific attention since lack of recognition of the device (e.g., tugging it, or trivializing its management) could lead to the worsening of clinical outcomes (e.g., infections or detaching of the driveline) and problems in the patient’s identity as heard in the interviews. This process of dehumanization could represent an avoidance coping strategy through which people attempt to protect themselves from emotional suffering by attributing less human characteristics to the illness condition (Capozza et al., 2016). This “defensive mechanism” typically occurs in the medical field, where patients are treated as mechanical systems and the focus of the physicians is often on a specific part of the body, often the sick one, without consideration of the patient’s perspective about the illness and their mental state (Capozza et al., 2016). The dehumanization could be used by those caregivers who are less empathic and able to detect other people’s complex emotions and elaborate thoughts (Cameron et al., 2016). This mechanism has previously been studied in patients with obstructive sleep apnea syndrome following continuous positive airway pressure therapy and showed similar characteristics in that the machine was considered an extension of themselves and part of their daily life (Rapelli et al., 2022b).

Family caregivers seemed to play a crucial role in successful LVAD outcomes (Cook et al., 2017; McIlvennan et al., 2021) by assisting the patients with daily dressing changes and the maintenance of the device, as well as prompting intervening-following the emergency protocols at home-in case of need. Family caregivers’ competencies were acquired and developed in the hospital through intensive education and training, but the ride home could still cause feelings of self-doubt. In fact, the transition from hospital to home represented another important challenge for the caregivers, as they had to adjust to their new roles, responsibilities, and lifestyle modifications (Marcuccilli et al., 2014). Another aspect was the caregiver’s burden, already recognized in several studies as a critical element that could affect the health of the patients and of the caregivers themselves (Cicolini et al., 2016; Rapelli et al., 2020; Waldenburger et al., 2020). In this study, too, the burden of caregiving emerged. According to other studies (Marcuccilli et al., 2014; Cicolini et al., 2016; Magasi et al., 2019), patients were afraid of overburdening caregivers and being a burden, they feared that they will get tired and get sick themselves. The self-perceived burden is associated with decreased help-seeking, decreased quality of life, as well as psychological and existential distress among patients with LVAD and their caregivers (Zimmermann et al., 2021; Atila and Ozsaker, 2022). Caregivers likewise feared that they will fail and be swallowed up by stress and domestic chores. These findings indicate the need for increased psychosocial screening and intervention screening and intervention to help patients manage feelings of diminished autonomy and loss of role and caregivers to cope with role change with more information and awareness.

The LVAD also generated reflections on the meaning of life and bioethical aspect. In fact, both the patients and their caregivers talked about their opposing thoughts and feeling about the opportunity to be reborn and about the end-of-life stage showing mixed feelings: gratitude to God and/or to science progress, but also feel themselves as inappropriate and reprehensible to such an extent to feel “against nature.” The topic of the spiritual domain was previously been little explored among patients with LVAD (Grady et al., 2001), discussing the increase of faith in God during this period of illness, but the topic has never been explored among caregivers who use spirituality to cope with the uncertainty of the period. This spiritual theme also strongly emerged in relation to heart transplantation and waiting for a new heart: Patients and caregivers showed feelings of hope and a concurrent sense of helplessness about this expectation. In general, concerning this last theme, the sense of passivity that patients and caregivers showed during the implantation and in the phases to come, such as waiting for the heart transplant, emerged, to say of an impotent resignation and mastery of events and a total dependence on medicine or the will of God. Although the LVAD implant was a free choice of the patient or his/her family (if the choice of the implant is derived from the caregiver in situations where the patient is not conscious to express his opinion, for example in a coma) it appeared as a choice “dropped from the above” in which patient and caregiver feel little protagonist of this choice and unable to control and manage it.

**Figure 1:**
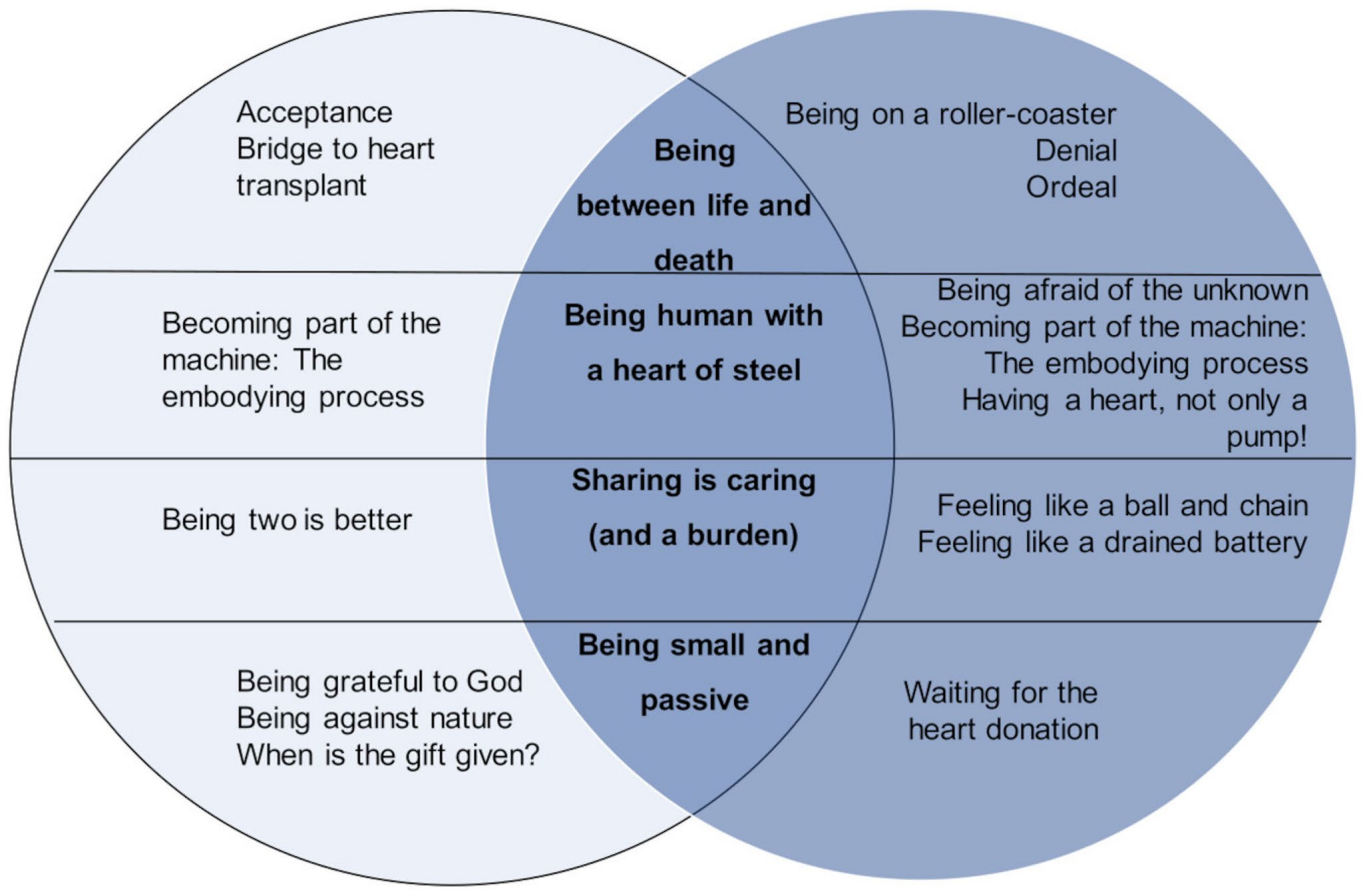
The LVAD experience of patients and their caregivers.

The final picture is of a patient who strongly experiences ambivalence with respect to the device insofar as it is a life-saving device, but at the same time difficult to manage, in fact it also emerges that the presence of others is fundamental, but also a source of concern with respect to caregiver burden issues. Moreover, the patient is also involved in the new redefinition of self in terms of body perception and identity, as the presence of an artificial heart opens up ethical and value debates (Figure 1).

## Limitations and strengths

The findings presented in this study are limited for several reasons: First, the patient sample consisted only of white men recruited from a single medical setting, limiting generalizability to other LVAD patients; second all patients have a BTT-LVAD, so the experience of LVAD as destination therapy (DT) was not explored in this study; third, as the aim of this study is to collect LVAD experience from both patient and caregiver, the experience of patient with no delegated caregiver was not considered. Although in other contexts it is possible to be single, in Italy living alone precludes LVAD consideration. Furthermore, as our study is performed with 12 individual interviews coming from 6 dyads, future studies with more participants could be crucial to confirm our results. Moreover, a second phase of the study interviewing participants and their caregiver after the return at home, could add specific information on how they could change their perspectives about LVAD management in the long-term. In this study, the patient’s and caregiver’s feedback on final findings are not collected, but in future studies, a member check validation or a stakeholder survey could be useful in order to implement an effective psychological intervention in which stakeholders are engaged. As a strength we can recognize the inclusion of different close relatives, which has allowed triangulation of data through the narration of multiple informants; furthermore the inclusion of patients and caregivers and the dyadic perspective adopted in the data analysis. Moreover, although the growing literature on the LVAD management at home, this study adds a specific contribution to the initial adjustment to the device. In fact, this phase could be crucial for the patient’s chronic adjustment and as already demonstrated in other studies, the initial psychological reaction to LVAD could be a strong predictor of the patient’s quality of life 1 year after the implant (Brouwers et al., 2015; Bidwell et al., 2017; Casida et al., 2018). Moreover, patients and caregivers reported many worries and thoughts never investigated in the literature on LVAD that raise ethical and social issues of the psychological process adaptation that could be addressed in an effective psychological intervention.

In conclusion, given its qualitative nature, this study gives voice to the patients’ and caregivers’ lived experiences with LVAD, thus allowing the recognition and deep investigation of their feelings, emotions, and worries on several topics emerged, underlining the dyadic nature of emotional adjustment to LVAD and the impact in patients and caregivers’ life. These could guide the healthcare providers in the assessment of the adjustment and health status of patients and their caregivers to LVAD.

Based on our findings, we recommend that clinical teams should:

- Help both patients and caregivers to discuss the emotional and interpersonal impact of LVAD;
- Help both patients and caregivers to discuss their roles at home regarding device management so as to clarify mutual role expectations;
- Promote patient and caregiver’s engagement in meaningful life roles and activities and
- Better prepare and support caregivers for this role throughout the caregiving experience;
- Recognize both conscious and unconscious signs of psychological rejection of LVAD by patients and caregivers (e.g., consider the LVAD as an extension of himself/herself or tug on the device);
- Discuss how much the LVAD implant is a forced choice or in line with patients’ and caregivers’ personal values;
- Promote adequate coping strategies during each phase of the adjustment process to LVAD;
- Assess both patients’ and caregivers’ quality of life, symptoms of depression and anxiety in different phases of LVAD adjustment;
- Promote disclosure of challenging issues in order to promptly identify problems and recognize when additional intervention and professional psychological support is needed.

## Data availability statement

The raw data supporting the conclusions of this article will be made available by the authors, without undue reservation.

## Ethics statement

The studies involving human participants were reviewed and approved by Comitato etico Università Cattolica del Sacro Cuore di Milano. The patients/participants provided their written informed consent to participate in this study.

## Author contributions

GR, EG, SD, and AB contributed to the development of the study, analysis of the results, and writing of the manuscript. MP, AP, GP, and GC contributed to the development of the study and writing of the manuscript. All authors contributed to the article and approved the submitted version.

## Funding

This research was funded by the Italian Ministry of Health.

## Conflict of interest

The authors declare that the research was conducted in the absence of any commercial or financial relationships that could be construed as a potential conflict of interest.

## Publisher’s note

All claims expressed in this article are solely those of the authors and do not necessarily represent those of their affiliated organizations, or those of the publisher, the editors and the reviewers. Any product that may be evaluated in this article, or claim that may be made by its manufacturer, is not guaranteed or endorsed by the publisher.

## Footnotes

^1^The first author is a female psychologist (MD and Ph.D). At the time of the study, she worked as a doctoral student and psychologist in the hospital where the study took place. She had specific training in conducting interviews. Before the study, no relationship was established between the psychologist and participants.

^2^CR comprises multidisciplinary activities, including as-supervised physical training to restore functional independence, as well as psychological/neuropsychological assessment, intervention, and support for patients and their caregivers.
